# The kidneys matter

**DOI:** 10.1007/s00424-022-02737-0

**Published:** 2022-07-27

**Authors:** Johannes Loffing, Francois Verrey, Carsten A. Wagner

**Affiliations:** 1 National Center of Competence in Research NCCR Kidney, CH, University of Zurich, Zurich, Switzerland; 2 Institutes of Anatomy, University of Zurich, Zurich, Switzerland; 3 Institutes of Physiology, University of Zurich, Winterthurerstrasse 190, CH-8057 Zurich, Switzerland

More than 150 years ago, the French physiologist Claude Bernard formulated his theory of the “milieu de l’intérieur” writing:

*The fixity of the milieu supposes a perfection of the organism such that the external variations are at each instant compensated for and equilibrated.... All of the vital mechanisms, however varied they may be, have always one goal, to maintain the uniformity of the conditions of life in the internal environment .... The stability of the internal environment is the condition for the free and independent life.* (cited after [7]).

The central point of this theory is that organ and cell function need a stable environment. Almost 100 years later, the American nephrologist Homer Smith laid out in “From fish to philosopher” his view how the kidneys were central to the evolution of humans from organisms living in a salt-water environment by regulating central components of the internal homeostasis [14].

Both the role of the kidney in controlling and maintaining the constancy of the internal environment as well as the concept that kidneys were shaped to allow living in changing environments are nowadays integrative parts of our current understanding of this organ. Arguably, the kidneys are central organs for controlling and regulating the homeostasis of electrolytes, metabolites, and processes critical for normal function of most organs. Normal kidney function maintains the capacity of blood to transport oxygen and carbon dioxide through erythropoietin controlled erythropoiesis, regulates blood pressure by controlling salt, potassium, and water balance, modulates acid–base and mineral homeostasis, or contributes to overall metabolism through gluconeogenesis, just to name a few functions.

Kidney disease affects worldwide 10–12% of the population and its prevalence is expected to rather increase with the aging of populations and the rise of conditions such as diabetes and hypertension that can cause or accelerate kidney disease [4]. At the same time, recent years have marked an enormous progress in various fields related to a better understanding of the morphology, the genetics, and the physiology of kidneys and brought new insights into the cause and progress of kidney disease.

Novel drugs such as SGLT2 inhibitors, improved mineralocorticoid receptor antagonists, and biologicals targeting various hormones, cytokines, and receptors are currently revolutionizing care of patients with kidney disease. Obviously, the development of these drugs is rooted in many decades of basic and clinical research. A series of new technological developments and improvement in fields such as microscopy and imaging, the rise of population-based genetics, and genomics as well as the faster and more affordable possibilities for whole genome or exome sequencing, single cell transcriptomics, and improved proteome or metabolome analyses, modeling of genetic and other data, refined animal models, and the development of new models such as iPSCs have accelerated research and open new avenues for unbiased research as well as for formulating new hypotheses and putting them at test.

Notably, these innovations and transitions have made research often depending on consortia with complementary competences and expertise. On this background, the Swiss National Science Foundation together with the Swiss Federal Council decided in 2009 to fund a Switzerland-wide research network as a National Center of Competence (NCCR) named Kidney.CH where CH stands both for Control of Homeostasis and for Switzerland. During 12 years, nephrologists and basic researchers from various fields collaborated to tackle fundamental questions about how the kidneys work, and how these processes are altered in and contribute to kidney disease.

This special issue of *Pflügers Archiv – European Journal of Physiology* assembles a series of reviews from researchers who were participants of the NCCR Kidney.CH as well as from several international experts that were part of the review panel, the international advisory board, or collaborated with groups of the NCCR Kidney.CH. All reviews address various topics how kidneys control and maintain aspects of homeostasis and how some of these mechanisms also contribute to disease processes.

In a first group of reviews, Kurtcuoglu and Edwards describe recent advances in imaging renal vasculature and modeling blood flow and tissue oxygenation [5], while Devuyst and Bochud discuss new insights into the contribution of rare and common genetic variants to renal function and risk to develop kidney disease [3]. Wenger and colleagues as well as Broeker and Kurtz examine the origin, characteristics, and plasticity of cells producing erythropoietin or renin, respectively [1, 2]. Last, Hall and de Seigneux review novel concepts how renal metabolism is altered during acute kidney injury [8].

The role of the kidney in the control of electrolyte and water homeostasis is highlighted in a second section. Hummler et al. examine glucocorticoid signaling in kidney and adrenal gland [16], while Feraille and Hoorn analyze the current state of understanding how renal handling of water controls water balance [6]. McDonough and Fenton review mechanisms of extrarenal and renal sensing of potassium and its transport in the kidney [9] followed by a discussion by Pearce, Nesterov, and Korbmacher on the function of the distal segments of the nephron in sodium transport [10].

The last set of reviews focuses on function of the kidney that relate to mineral and acid–base balance. Novel insights into mechanisms underlying renal calcium and magnesium transport are described by Houillier and colleagues and by de Baaij, Hoenderop, and colleagues, respectively [11, 15]. Imenez Silva and Mohebbi summarize recent concepts how the kidney contributes to acid–base homeostasis and how acidosis may impact on kidney disease progression. The impact of nutritional phosphate on kidneys, the regulation of phosphate balance by the kidneys, and the question whether phosphate may have toxic effects are discussed by Rubio-Aliaga and Krapf [12]. Finally, Rudloff, Jahnen-Dechent, and Huynh-Do describe a role of fetuin-A as a regulator of systemic and tissue calcification and its potential as novel therapeutic [13].

Collectively, all these reviews reinforce the notion that kidneys have evolved during evolution as a central organ to control the milieu interieur enabling normal and complex cellular and organ function and as a condition to allow to adapt to ever changing environmental and life-style factors as described by Claude Bernard and Homer Smith. Some of the renal mechanisms, however, also limit our capacity to cope with modern life-style as exemplified by the impact of diets rich in salt and poor in potassium. Understanding these limitations helps to device strategies to prevent disease or to develop novel therapeutic strategies for patients with kidney disease.

## References

[1] Broeker KAE, Schrankl J, Fuchs MAA, Kurtz A (2022). Flexible and multifaceted: the plasticity of renin-expressing cells. Pflugers Arch.

[2] Dahl SL, Bapst AM, Khodo SN, Scholz CC, Wenger RH (2022). Fount, fate, features, and function of renal erythropoietin-producing cells. Pflugers Arch.

[3] Devuyst O, Bochud M, Olinger E (2022). *UMOD* and the architecture of kidney disease. Pflügers Arch.

[4] Eckardt KU, Coresh J, Devuyst O, Johnson RJ, Kottgen A, Levey AS, Levin A (2013). Evolving importance of kidney disease: from subspecialty to global health burden. Lancet.

[5] Edwards A, Kurtcuoglu V (2022). Renal blood flow and oxygenation. Pflugers Arch.

[6] Feraille E, Sassi A, Olivier V, Arnoux G, Martin PY (2022). Renal water transport in health and disease. Pflugers Arch.

[7] Gross CG (2009). Three before their time: neuroscientists whose ideas were ignored by their contemporaries. Exp Brain Res.

[8] Hall AM, de Seigneux S (2022). Metabolic mechanisms of acute proximal tubular injury. Pflugers Arch.

[9] McDonough AA, Fenton RA (2022). Potassium homeostasis: sensors, mediators, and targets. Pflugers Arch.

[10] Pearce D, Manis AD, Nesterov V, Korbmacher C (2022). Regulation of distal tubule sodium transport: mechanisms and roles in homeostasis and pathophysiology. Pflugers Arch.

[11] Prot-Bertoye C, Lievre L, Houillier P (2022) The importance of kidney calcium handling in the homeostasis of extracellular fluid calcium. Pflügers Arch. 10.1007/s00424-022-02725-4 10.1007/s00424-022-02725-4 35842482

[12] Rubio-Aliaga I, Krapf R (2022). Phosphate intake, hyperphosphatemia, and kidney function. Pflugers Arch.

[13] Rudloff S, Jahnen-Dechent W, Huynh-Do U (2022). Tissue chaperoning-the expanded functions of fetuin-A beyond inhibition of systemic calcification. Pflugers Arch.

[14] Smith HW (1953). From fish to philosopher.

[15] Tholen LE, Hoenderop JGJ, de Baaij JHF (2022). Mechanisms of ion transport regulation by HNF1beta in the kidney: beyond transcriptional regulation of channels and transporters. Pflugers Arch.

[16] Verouti S, Hummler E, Vanderriele PE (2022). Role of glucocorticoid receptor mutations in hypertension and adrenal gland hyperplasia. Pflugers Arch.

